# Supplementary material for: Drug repurposing for aging research using model organisms
Source: Aging Cell. 2017 Jun 16;16(5):1006–15. doi: 10.1111/acel.12626 (PMC5595691; doi:10.1111/acel.12626)
Supplement: Supplementary file 7 — Data S1 Zip‐Archive of all report cards. [file ACEL-16-1006-s007.zip › RC_PEG.pdf]

PEG

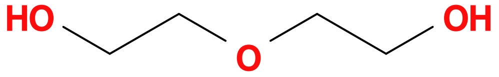

Database identifiers

ChEMBLCompound	CHEMBL1235226
CHEBI	46807
ZINC	ZINC01668756
eMolecules	475220

Ranking

	Rank	Score
Drosophila	547/697	0.204
C. elegans	586/591	0.0

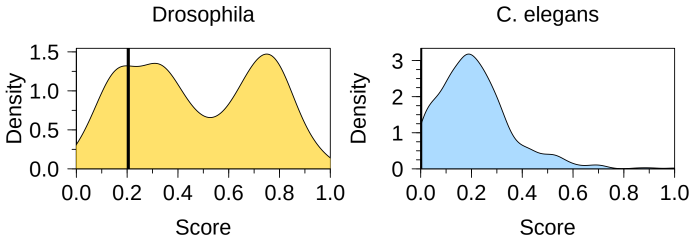

	Ageing implication	Domain conservation	Binding site conservation	Binding affinity	Bioavailability	Lipinski	Promiscuity	Purchasability	Drug approval	**Total**
Drosophila	1.0	0.982	1.0	0.344	(0.9)	0.0	-0.2	0.1	0.0	0.204
C. elegans	1.0	0.96	1.0	0.344	0.231	0.0	-0.2	0.1	0.0	0.0

Names

- **diethylene glycol**
- 1,5-Dihydroxy-3-oxapentane
- 2,2'-dihydroxydiethyl ether
- 2,2'-Oxydiethanol
- 2-(2-hydroxyethoxy)ethanol
- beta,beta'-dihydroxydiethyl ether
- bis(2-hydroxyethyl) ether
- bis(beta-hydroxyethyl) ether
- Di(hydroxyethyl)ether
- Diethylenglykol

Roles

ChEBI entry 46807 has no roles

Status

Approved drug (according to ChEMBL)	No
Number of Rule of 5 violations	0
Binding affinity to original target in log units (RF-Score prediction)	4.36
Burns *C. elegans* bioavailability prediction	-3.21

Compound Target Characteristics

Menin

Best gene implication in ageing for this target family came from gene Q8SXU2 annotated in UniProt release 2014_02. Annotation GO 8340 (determination of adult lifespan) was Inferred from Mutant Phenotype

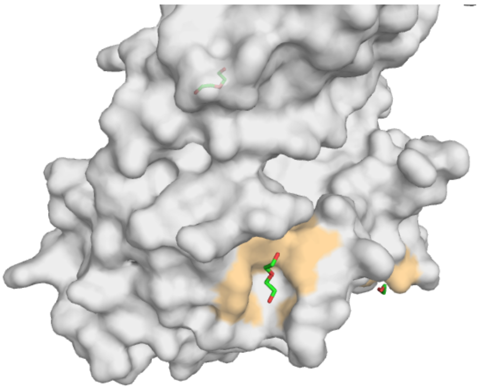

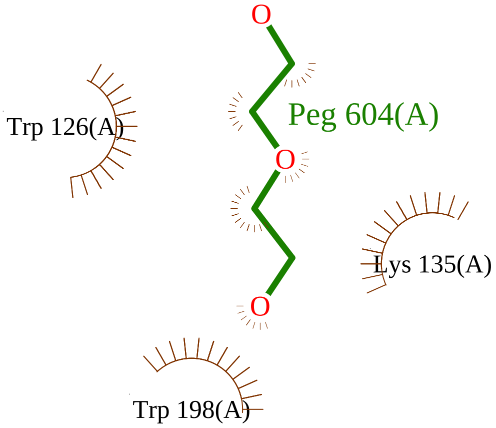

protein	amino acids contacts (binding site)
PDB:4gpq:chainA:O00255	H F V N T W S K W Y D G E K Y Y
PDB:4gq3:chainA:O00255	H F V N T W S K W Y D G E K Y Y
PDB:4og3:chainA:O00255	H F V N T W S K W Y D G E K Y Y
PDB:4og4:chainA:O00255	H F V N T W S K W Y D G E K Y Y
PDB:4og5:chainA:O00255	H F V N T W S K W Y D G E K Y Y
PDB:4og6:chainA:O00255	H F V N T W S K W Y D G E K Y Y
PDB:4og7:chainA:O00255	H F V N T W S K W Y D G E K Y Y
tr:E7ET29:E7ET29_HUMAN	H F V N T W S K W Y D G E K Y Y
tr:E7EN32:E7EN32_HUMAN	H F V N T W S K W Y D G E K Y Y
sp:O00255:MEN1_HUMAN	H F V N T W S K W Y D G E K Y Y
sp:Q9WVR8:MEN1_RAT	H F V N T W S K W Y D G E K Y Y
sp:O88559:MEN1_MOUSE	H F V N T W S K W Y D G E K Y Y
tr:F8WHD9:F8WHD9_MOUSE	H F V N T W S K W Y D G E K Y Y
tr:Q8SXU2:Q8SXU2_DROME	L S T G S W I K W Y D G K E Y Y
tr:Q7KXY9:Q7KXY9_DROME	L S T G S W I K W Y D G K E Y Y
tr:Q9VM47:Q9VM47_DROME	L S T G S W I K W Y D G K E Y Y

	whole protein		domain-based		contact-based	
protein	ident	simil	ident	simil	ident	simil
PDB:4gpq:chainA:O00255	1.0	1.0	1.0	1.0	1.0	1.0
PDB:4gq3:chainA:O00255	1.0	1.0	1.0	1.0	1.0	1.0
PDB:4og3:chainA:O00255	0.99	1.0	0.99	1.0	1.0	1.0
PDB:4og4:chainA:O00255	0.99	1.0	0.99	1.0	1.0	1.0
PDB:4og5:chainA:O00255	0.99	1.0	0.99	1.0	1.0	1.0
PDB:4og6:chainA:O00255	1.0	1.0	1.0	1.0	1.0	1.0
PDB:4og7:chainA:O00255	1.0	1.0	1.0	1.0	1.0	1.0
tr:E7ET29:E7ET29_HUMAN	0.56	0.56	0.56	0.56	1.0	1.0
tr:E7EN32:E7EN32_HUMAN	0.9	0.9	0.9	0.9	1.0	1.0
sp:O00255:MEN1_HUMAN	1.0	1.0	1.0	1.0	1.0	1.0
sp:Q9WVR8:MEN1_RAT	0.96	0.98	0.96	0.98	1.0	1.0
sp:O88559:MEN1_MOUSE	0.96	0.98	0.96	0.98	1.0	1.0
tr:F8WHD9:F8WHD9_MOUSE	0.95	0.97	0.95	0.97	1.0	1.0
tr:Q8SXU2:Q8SXU2_DROME	0.36	0.64	0.37	0.65	0.5	0.58
tr:Q7KXY9:Q7KXY9_DROME	0.32	0.6	0.34	0.64	0.5	0.58
tr:Q9VM47:Q9VM47_DROME	0.32	0.59	0.34	0.64	0.5	0.58

Mnn1 (FBgn0031885) associated phenotypes

DNA repair defective, chemical sensitive, conditional, heat sensitive, lethal - all die before end of

P-stage, melanotic mass phenotype, radiation sensitive, short lived, some die during pharate adult stage, stress response defective

(Information from FlyBase)

Superoxide dismutase [Cu-Zn]

Best gene implication in ageing for this target family came from gene P61851 annotated in UniProt release 2014_02. Annotation GO 8340 (determination of adult lifespan) was Inferred from Mutant Phenotype

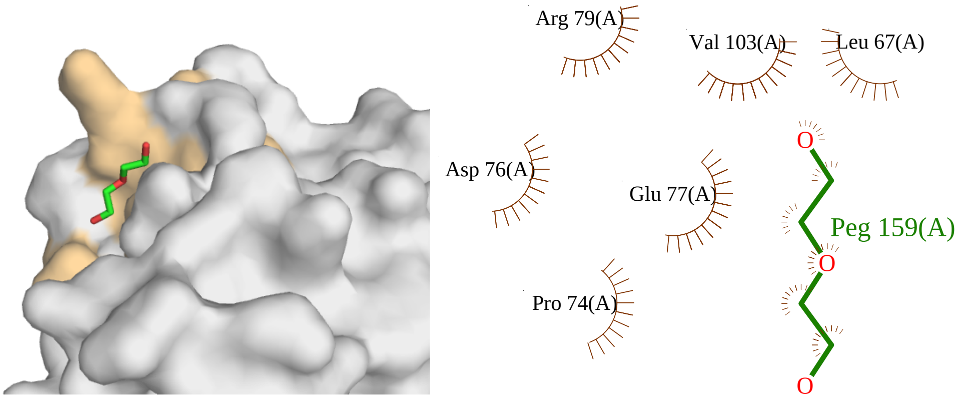

protein	amino acids contacts (binding site)
PDB:2xjl:chainA:P00441	L P D E R V
tr:H7BYH4:H7BYH4_HUMAN	L P D E R V
sp:P00441:SODC_HUMAN	L P D E R V
sp:P07632:SODC_RAT	H P D E R V
tr:Q6LDS4:Q6LDS4_RAT	H P D E R V
sp:P08228:SODC_MOUSE	H P D E R V
sp:P61851:SODC_DROME	Y P D E R K
tr:A1Z850:A1Z850_DROME	R P A E R V
tr:Q9BLY4:Q9BLY4_DROME	R P A E R V
tr:HORNH3:HORNH3_DROME	R P A E R V
tr:C6TP82:C6TP82_DROME	R P A E R V
tr:G5ECR5:G5ECR5_CAEEL	H P D S R L
sp:P34461:SODE_CAEEL	H P D S R L
sp:P00445:SODC_YEAST	F P D E R L

	whole protein		domain-based		contact-based	
protein	ident	simil	ident	simil	ident	simil
PDB:2xjl:chainA:P00441	0.95	0.97	0.95	0.97	1.0	1.0
tr:H7BYH4:H7BYH4_HUMAN	0.88	0.88	0.87	0.87	1.0	1.0
sp:P00441:SODC_HUMAN	1.0	1.0	1.0	1.0	1.0	1.0
sp:P07632:SODC_RAT	0.83	0.94	0.83	0.94	0.83	0.85
tr:Q6LDS4:Q6LDS4_RAT	0.82	0.93	0.82	0.93	0.83	0.85
sp:P08228:SODC_MOUSE	0.84	0.94	0.83	0.94	0.83	0.85
sp:P61851:SODC_DROME	0.61	0.84	0.6	0.85	0.67	0.79
tr:A1Z850:A1Z850_DROME	0.27	0.46	0.42	0.7	0.67	0.65
tr:Q9BLY4:Q9BLY4_DROME	0.27	0.46	0.42	0.7	0.67	0.65
tr:HORNH3:HORNH3_DROME	0.26	0.44	0.42	0.7	0.67	0.65
tr:C6TP82:C6TP82_DROME	0.26	0.44	0.42	0.7	0.67	0.65
tr:G5ECR5:G5ECR5_CAEEL	0.35	0.57	0.44	0.71	0.5	0.67
sp:P34461:SODE_CAEEL	0.35	0.57	0.44	0.71	0.5	0.67
sp:P00445:SODC_YEAST	0.54	0.83	0.55	0.84	0.67	0.92

Sod (FBgn0003462) associated phenotypes

calorie restriction conditional, chemical conditional, chemical sensitive, heat sensitive, increased cell

number, locomotor behavior defective, long lived, male limited, oxidative stress response defective, partially, partially lethal - majority die, progressive, radiation sensitive, reduced, short lived

(Information from FlyBase)

Sod (UniProt:P61851) annotation
Function: Destroys radicals which are normally produced within the cells and which are toxic to biological systems.
Cofactor:Cu cationNote=Binds 1 copper ion per subunit.;
Cofactor: Zn(2+)Note=Binds 1 zinc ion per subunit.;
Subunit: Homodimer.
Subcellular location: Cytoplasm.

(Information from UniProt)

Ccs (FBgn0010531) associated phenotypes
chemical sensitive, short lived

(Information from FlyBase)

Ccs (UniProt:A1Z850) annotation
Function: Destroys radicals which are normally produced within the cells and which are toxic to biological systems. (RuleBase:RU000393).
Cofactor: Cu cation Evidence=(RuleBase:RU000393); Note=Binds 1 copper ion per subunit. (RuleBase:RU000393);
Cofactor: Zn(2+) Evidence=(RuleBase:RU000393); Note=Binds 1 zinc ion per subunit. (RuleBase:RU000393);

(Information from UniProt)

Ccs (UniProt:Q9BLY4) annotation
Function: Destroys radicals which are normally produced within the cells and which are toxic to biological systems. (RuleBase:RU000393).
Cofactor: Cu cation Evidence=(RuleBase:RU000393); Note=Binds 1 copper ion per subunit. (RuleBase:RU000393);
Cofactor: Zn(2+) Evidence=(RuleBase:RU000393); Note=Binds 1 zinc ion per subunit. (RuleBase:RU000393);

(Information from UniProt)

CCS-RB (UniProt:H0RNH3) annotation
Function: Destroys radicals which are normally produced within the cells and which are toxic to biological systems. (RuleBase:RU000393).
Cofactor: Cu cation Evidence=(RuleBase:RU000393); Note=Binds 1 copper ion per subunit. (RuleBase:RU000393);
Cofactor: Zn(2+) Evidence=(RuleBase:RU000393); Note=Binds 1 zinc ion per subunit. (RuleBase:RU000393);

(Information from UniProt)

CCS-RA (UniProt:C6TP82) annotation
Function: Destroys radicals which are normally produced within the cells and which are toxic to biological systems. (RuleBase:RU000393).
Cofactor: Cu cation Evidence=(RuleBase:RU000393); Note=Binds 1 copper ion per subunit. (RuleBase:RU000393);
Cofactor: Zn(2+) Evidence=(RuleBase:RU000393); Note=Binds 1 zinc ion per subunit. (RuleBase:RU000393);

(Information from UniProt)

sod-4 (UniProt:G5ECR5) annotation

Function: Destroys radicals which are normally produced within the cells and which are toxic to biological systems. (RuleBase:RU000393).

Cofactor: Cu cation Evidence=(RuleBase:RU000393); Note=Binds 1 copper ion per subunit. (RuleBase:RU000393);

Cofactor: Zn(2+) Evidence=(RuleBase:RU000393); Note=Binds 1 zinc ion per subunit. (RuleBase:RU000393);

(Information from UniProt)

sod-4 (UniProt:P34461) annotation

Function: Destroys radicals which are normally produced within the cells and which are toxic to biological systems.

Cofactor:Cu cationNote=Binds 1 copper ion per subunit. ;

Cofactor: Zn(2+)Note=Binds 1 zinc ion per subunit. ;

Subcellular location: Isoform 1: Secreted, extracellular space.

Subcellular location: Isoform 2: Membrane ; Peripheral membrane protein

Tissue specificity: Isoform 2 is preferentially expressed in eggs.

(Information from UniProt)

Phosphoenolpyruvate carboxykinase, cytosolic [GTP]

Best gene implication in ageing for this target family came from gene Q9Z2V4 via mapping the annotation from Ensembl ENSMUSG00000027513 via mapping the annotation from EntrezGene 18534 via mapping the annotation from GenAgeModels 0497 annotated in GenAge release 17.

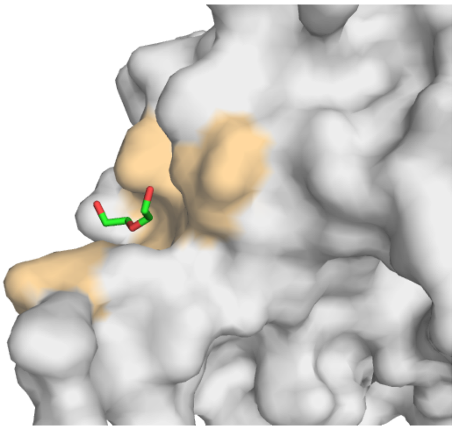

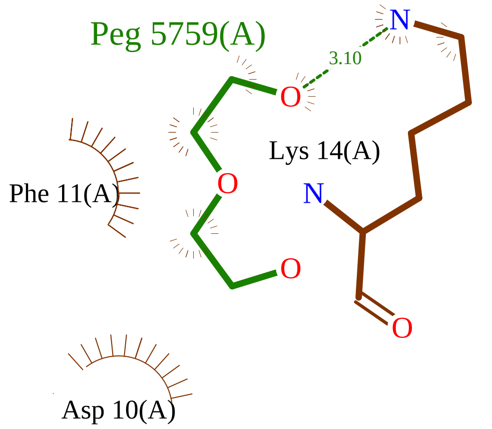

protein	amino acids contacts (binding site)
PDB:2rk8:chainA:P07379	N D F K Q
sp:P35558:PCKGC_HUMAN	N N L K Q
sp:P07379:PCKGC_RAT	N D F K Q
sp:Q9Z2V4:PCKGC_MOUSE	N D F K Q
tr:Q7JXB5:Q7JXB5_DROME	G L L S Q
sp:P20007:PCKG_DROME	Q I I P Q
tr:Q9TYQ8:Q9TYQ8_CAEEL	P Q F P T

protein	whole protein ident	simil	domain-based ident	simil	contact-based ident	simil
PDB:2rk8:chainA:P07379	1.0	1.0	1.0	1.0	1.0	1.0
sp:P35558:PCKGC_HUMAN	0.91	0.98	0.89	0.98	0.6	0.9
sp:P07379:PCKGC_RAT	1.0	1.0	1.0	1.0	1.0	1.0
sp:Q9Z2V4:PCKGC_MOUSE	0.98	0.99	0.98	0.99	1.0	1.0
tr:Q7JXB5:Q7JXB5_DROME	0.62	0.85	0.51	0.78	0.2	0.32
sp:P20007:PCKG_DROME	0.61	0.84	0.51	0.76	0.2	0.37
tr:Q9TYQ8:Q9TYQ8_CAEEL	0.22	0.59	0.22	0.61	0.2	0.55

Pepck (UniProt:P20007) annotation

Function: Catalyzes the conversion of oxaloacetate (OAA) to phosphoenolpyruvate (PEP), the rate-limiting step in the metabolic pathway that produces glucose from lactate and other precursors derived from the citric acid cycle.
Cofactor: Mn(2+)Note=Binds 1 Mn(2+) ion per subunit. ;
Pathway: Carbohydrate biosynthesis; gluconeogenesis.
Subunit: Monomer.

(Information from UniProt)

Poly [ADP-ribose] polymerase 1

Best gene implication in ageing for this target family came from gene Q921K2 via mapping the annotation from Ensembl ENSMUSG00000026496 via mapping the annotation from EntrezGene 11545 via mapping the annotation from GenAgeModels 1010 annotated in GenAge release 17.

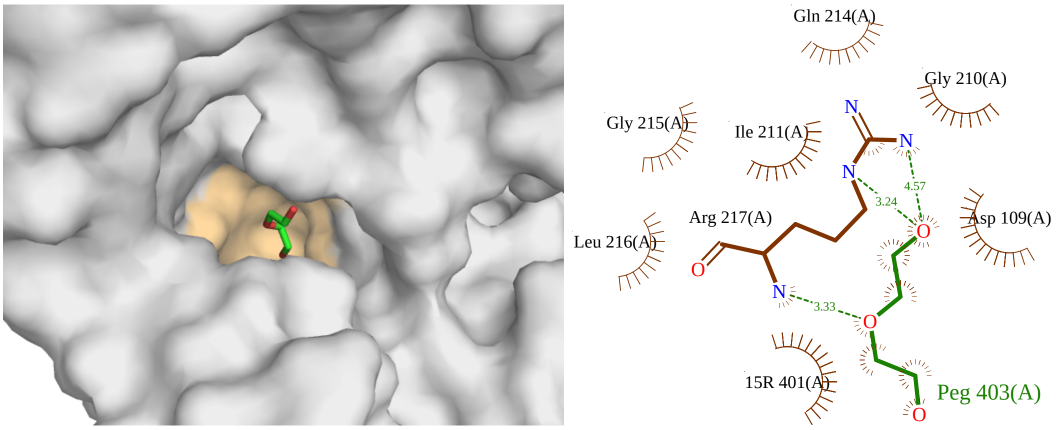

protein	amino acids contacts (binding site)
PDB:4hhy:chainA:P09874	D S G I Q G L R
sp:P09874:PARP1_HUMAN	D S G I Q G L R
sp:P27008:PARP1_RAT	D S G I Q G L R
tr:Q921K2:Q921K2_MOUSE	D S G I Q G L R
sp:P35875:PARP_DROME	E S G I H G L R
sp:Q9N4H4:PME1_CAEEL	D S G I Q G L R

protein	whole protein ident	simil	domain-based ident	simil	contact-based ident	simil
PDB:4hhy:chainA:P09874	1.0	1.0	1.0	1.0	1.0	1.0
sp:P09874:PARP1_HUMAN	1.0	1.0	1.0	1.0	1.0	1.0
sp:P27008:PARP1_RAT	0.92	0.97	0.95	0.99	1.0	1.0
tr:Q921K2:Q921K2_MOUSE	0.92	0.98	0.96	0.99	1.0	1.0
sp:P35875:PARP_DROME	0.41	0.77	0.54	0.85	0.75	0.92
sp:Q9N4H4:PME1_CAEEL	0.29	0.63	0.44	0.8	1.0	1.0

Parp (FBgn0010247) associated phenotypes

aging defective, heat sensitive, heat stress response defective, immune response defective, increased cell death, lethal - all die before end of pupal stage, non-enhancer of variegation, non-suppressor of

variegation, planar polarity defective, some die during pupal stage

(Information from FlyBase)

Parp (UniProt:P35875) annotation

Function: Poly[ADP-ribose] polymerase modifies various nuclear proteins by poly(ADP-ribosyl)ation. The modification is dependent on DNA and is involved in the regulation of various important cellular processes such as differentiation, proliferation, and tumor transformation and also in the regulation of the molecular events involved in the recovery of cell from DNA damage. Plays a fundamental role in organizing chromatin on a global scale; isoform e autoregulates Parp transcription by influencing the chromatin structure of its heterochromatic environment. (PubMed:12183365).
Subcellular location: Nucleus ECO:0000255—PROSITE- ProRule:PRU00264, PubMed:12183365). Nucleus, nucleolus (PubMed:12183365). Note=Highly enriched in nucleoli, heterochromatic chromosomal regions, and diverse euchromatic sites in the cells of most embryonic and adult tissues.
Tissue specificity: Expressed in adult female oocytes, anal plates of stage 12 embryos and in cells around the central nervous system in later embryos. (PubMed:9565614).
Developmental stage: Expressed both maternally and zygotically in embryos, pupae and adults. Expression is highest in embryos. (PubMed:12183365, PubMed:9565614).
Miscellaneous: The ADP-D-ribosyl group of NAD(+) is transferred to an acceptor carboxyl group on a histone or the enzyme itself, and further ADP-ribosyl groups are transferred to the 2'-position of the terminal adenosine moiety, building up a polymer with an average chain length of 20-30 units.

(Information from UniProt)

pme-1 (UniProt:Q9N4H4) annotation

Function: Poly[ADP-ribose] polymerase modifies various nuclear proteins by poly(ADP-ribosyl)ation, a post-translational modification synthesized after DNA damage that appears as an obligatory step in a detection/signaling pathway leading to the reparation of DNA strand breaks and programmed cell death. Involved in protection of the genome against mutations, probably via some participation in DNA repair. (PubMed:12600937).
Subcellular location: Nucleus ECO:0000305.
Developmental stage: Predominantly expressed at early embryonic stages and later in L4 and adult stages. (PubMed:12145714).

(Information from UniProt)

Neural cell adhesion molecule 1

Best gene implication in ageing for this target family came from gene F1LUV9 via mapping the annotation from RGD 67378 annotated in RGD 2014-03-11. Annotation GO 7568 (aging) was Inferred from Direct Assay

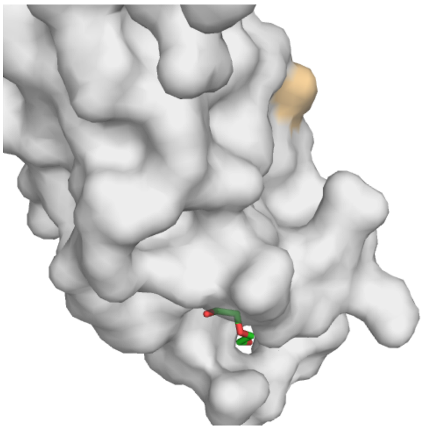

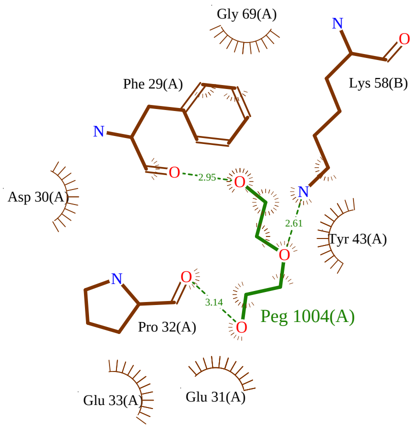

protein	amino acids contacts (binding site)												
PDB:2e3v:chainA:P13591	F	D	E	P	E	A	L	K	Y	K	G	L	N
PDB:2e3v:chainB:P13591	F	D	E	P	E	-	L	K	Y	K	G	L	N
tr:R4GMN9:R4GMN9_HUMAN	F	D	E	P	E	A	L	K	Y	K	G	L	N
tr:H7BYX6:H7BYX6_HUMAN	F	D	E	P	E	A	L	K	Y	K	G	L	N
sp:P13591:NCAM1_HUMAN	F	D	E	P	E	A	L	K	Y	K	G	L	N
tr:S4R389:S4R389_HUMAN	F	D	E	P	E	A	L	K	Y	K	G	L	N
tr:F1LUV9:F1LUV9_RAT	F	D	E	P	E	A	L	K	Y	K	G	L	N
tr:F1LNY3:F1LNY3_RAT	F	D	E	P	E	A	L	K	Y	K	G	L	N
tr:E9Q589:E9Q589_MOUSE	F	D	E	P	E	A	L	K	Y	K	G	L	N
tr:E9QB01:E9QB01_MOUSE	F	D	E	P	E	A	L	K	Y	K	G	L	N
tr:Q058Z9:Q058Z9_DROME	I	Q	T	P	R	M	Y	G	Y	A	K	R	N
tr:D0Z750:D0Z750_DROME	I	Q	T	P	R	M	Y	G	Y	A	K	R	N
tr:Q59E14:Q59E14_DROME	I	Q	T	P	R	M	Y	G	Y	A	K	R	N
tr:Q8T3E5:Q8T3E5_CAEEL	F	E	S	D	I	Y	M	E	L	A	-	V	N
tr:Q19128:Q19128_CAEEL	F	E	S	D	I	Y	M	E	L	A	-	V	N
tr:Q8MQ86:Q8MQ86_CAEEL	F	E	S	D	I	Y	M	E	L	A	-	V	N

	whole protein		domain-based		contact-based	
protein	ident	simil	ident	simil	ident	simil
PDB:2e3v:chainA:P13591	0.97	0.99	0.97	0.99	1.0	1.0
PDB:2e3v:chainB:P13591	0.98	0.99	0.98	0.99	0.92	0.83
tr:R4GMN9:R4GMN9_HUMAN	0.69	0.7	1.0	1.0	1.0	1.0
tr:H7BYX6:H7BYX6_HUMAN	0.7	0.75	0.55	0.59	1.0	1.0
sp:P13591:NCAM1_HUMAN	1.0	1.0	1.0	1.0	1.0	1.0
tr:S4R389:S4R389_HUMAN	0.92	0.92	1.0	1.0	1.0	1.0
tr:F1LUV9:F1LUV9_RAT	0.92	0.96	0.93	0.97	1.0	1.0
tr:F1LNY3:F1LNY3_RAT	0.94	0.97	0.93	0.98	1.0	1.0
tr:E9Q589:E9Q589_MOUSE	0.78	0.83	0.92	0.97	1.0	1.0
tr:E9QB01:E9QB01_MOUSE	0.94	0.97	0.92	0.97	1.0	1.0
tr:Q058Z9:Q058Z9_DROME	0.08	0.26	0.11	0.34	0.23	0.53
tr:D0Z750:D0Z750_DROME	0.1	0.33	0.11	0.34	0.23	0.53
tr:Q59E14:Q59E14_DROME	0.1	0.33	0.11	0.34	0.23	0.53
tr:Q8T3E5:Q8T3E5_CAEEL	0.18	0.53	0.14	0.46	0.15	0.41
tr:Q19128:Q19128_CAEEL	0.18	0.54	0.14	0.46	0.15	0.41
tr:Q8MQ86:Q8MQ86_CAEEL	0.17	0.52	0.14	0.46	0.15	0.41

Sucrase-isomaltase, intestinal

Best gene implication in ageing for this target family came from gene F1M792 via mapping the annotation from RGD 3675 annotated in RGD 2014-03-11. Annotation GO 7568 (aging) was Inferred from Expression Pattern

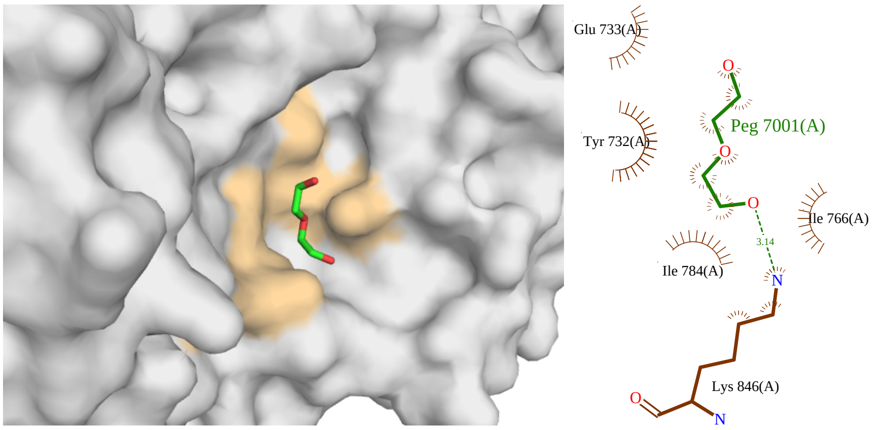

protein	amino acids contacts (binding site)
PDB:3lpp:chainA:P14410	Y E I I K
sp:P14410:SUIS_HUMAN	Y E I I K
tr:F1M792:F1M792_RAT	Y E T I K
tr:F8VQM5:F8VQM5_MOUSE	Y E T I K
tr:Q21750:Q21750_CAEEL	Y R R L E

	whole protein		domain-based		contact-based	
protein	ident	simil	ident	simil	ident	simil
PDB:3lpp:chainA:P14410	1.0	1.0	1.0	1.0	1.0	1.0
sp:P14410:SUIS_HUMAN	1.0	1.0	1.0	1.0	1.0	1.0
tr:F1M792:F1M792_RAT	0.77	0.92	0.71	0.91	0.8	0.79
tr:F8VQM5:F8VQM5_MOUSE	0.78	0.93	0.74	0.91	0.8	0.79
tr:Q21750:Q21750_CAEEL	0.17	0.37	0.26	0.7	0.2	0.64

Matrix metalloproteinase-9

Best gene implication in ageing for this target family came from gene D3ZYK8 via mapping the annotation from RGD 621320 annotated in RGD 2014-03-11. Annotation GO 7568 (aging) was Inferred from Expression Pattern

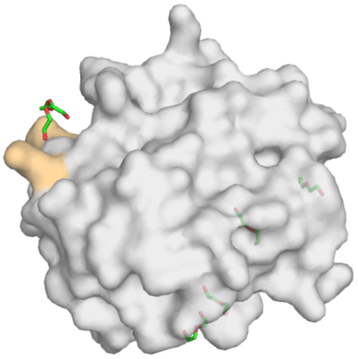

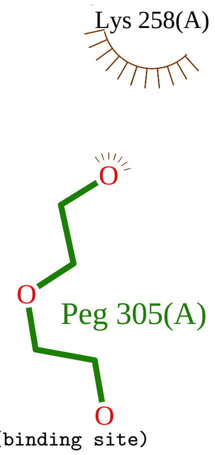

protein	amino acids contacts (binding site)
PDB:4h2e:chainA:P14780	N F T V A E D Y F K L V G Y E Y K
PDB:4hma:chainA:P14780	N F T V A E D Y F K L Q G Y E Y K
PDB:4jij:chainA:P14780	N F T V A E D Y F K L V G Y E Y K
sp:P14780:MMP9_HUMAN	N F T V A E D Y F K L Q G Y E Y K
tr:D3ZYK8:D3ZYK8_RAT	S F T V A E D Y F K L Q G Y E Y E
sp:P41245:MMP9_MOUSE	N F T V A E D Y F K L Q G Y E Y K
tr:G5EBU3:G5EBU3_CAEEL	S F S V R N D W F E N E A T N Y S

	whole protein		domain-based		contact-based	
protein	ident	simil	ident	simil	ident	simil
PDB:4h2e:chainA:P14780	0.99	0.99	0.99	0.99	1.0	1.0
PDB:4hma:chainA:P14780	0.99	1.0	0.99	1.0	0.94	0.94
PDB:4jij:chainA:P14780	0.98	0.99	0.98	0.99	1.0	1.0
sp:P14780:MMP9_HUMAN	1.0	1.0	1.0	1.0	0.94	0.94
tr:D3ZYK8:D3ZYK8_RAT	0.75	0.92	0.84	0.95	0.82	0.88
sp:P41245:MMP9_MOUSE	0.72	0.89	0.82	0.94	0.94	0.94
tr:G5EBU3:G5EBU3_CAEEL	0.18	0.45	0.33	0.67	0.29	0.55

Heterogeneous nuclear ribonucleoprotein L

Best gene implication in ageing for this target family came from gene Q0E914 annotated in UniProt release 2014_02. Annotation GO 8340 (determination of adult lifespan) was Inferred from Mutant Phenotype

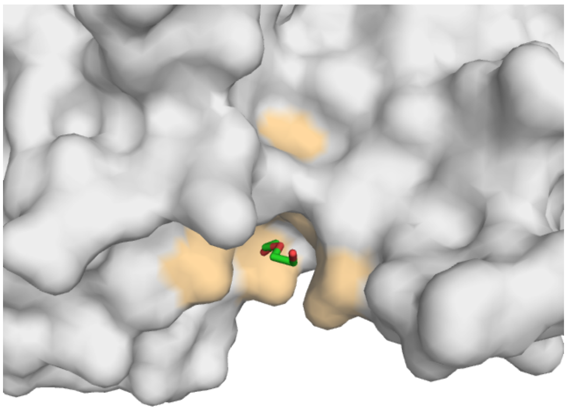

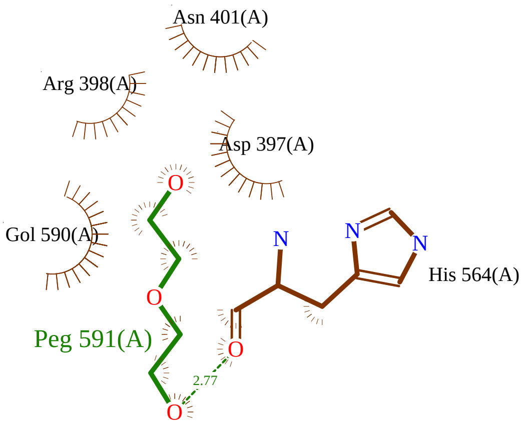

protein	amino acids contacts (binding site)					
PDB:3to8:chainA:P14866	D	R	N	N	H	Q
tr:Q6NTA2:Q6NTA2_HUMAN	D	R	N	N	H	Q
sp:P14866:HNRPL_HUMAN	D	R	N	N	H	Q
tr:Q5U1Y5:Q5U1Y5_RAT	D	R	N	N	H	Q
tr:F2Z3R2:F2Z3R2_RAT	D	R	N	N	H	Q
tr:F1LQ48:F1LQ48_RAT	D	R	N	N	H	Q
tr:G3UY38:G3UY38_MOUSE	D	R	N	N	H	Q
sp:Q8R081:HNRPL_MOUSE	D	R	N	N	H	Q
tr:Q0E914:Q0E914_DROME	D	K	N	N	H	P
tr:A1ZBN3:A1ZBN3_DROME	D	K	N	N	H	P
tr:Q8IHH3:Q8IHH3_DROME	D	K	N	N	H	P
tr:D5AEJ9:D5AEJ9_DROME	D	K	N	N	H	P
tr:Q7JMZ7:Q7JMZ7_DROME	D	K	N	N	H	P
tr:Q6NND8:Q6NND8_DROME	D	K	N	N	H	P
tr:Q95QR5:Q95QR5_CAEEL	D	M	N	N	H	P

	whole protein		domain-based		contact-based	
protein	ident	simil	ident	simil	ident	simil
PDB:3to8:chainA:P14866	0.99	0.99	0.99	0.99	1.0	1.0
tr:Q6NTA2:Q6NTA2_HUMAN	0.97	0.97	1.0	1.0	1.0	1.0
sp:P14866:HNRPL_HUMAN	1.0	1.0	1.0	1.0	1.0	1.0
tr:Q5U1Y5:Q5U1Y5_RAT	0.41	0.41	0.98	1.0	1.0	1.0
tr:F2Z3R2:F2Z3R2_RAT	0.98	0.99	0.98	1.0	1.0	1.0
tr:F1LQ48:F1LQ48_RAT	0.92	0.93	0.98	1.0	1.0	1.0
tr:G3UY38:G3UY38_MOUSE	0.77	0.77	0.98	1.0	1.0	1.0
sp:Q8R081:HNRPL_MOUSE	0.98	0.99	0.98	1.0	1.0	1.0
tr:Q0E914:Q0E914_DROME	0.15	0.28	0.34	0.67	0.67	0.84
tr:A1ZBN3:A1ZBN3_DROME	0.16	0.38	0.34	0.67	0.67	0.84
tr:Q8IHH3:Q8IHH3_DROME	0.16	0.38	0.34	0.67	0.67	0.84
tr:D5AEJ9:D5AEJ9_DROME	0.17	0.43	0.34	0.67	0.67	0.84
tr:Q7JMZ7:Q7JMZ7_DROME	0.25	0.49	0.34	0.67	0.67	0.84
tr:Q6NND8:Q6NND8_DROME	0.25	0.49	0.34	0.67	0.67	0.84
tr:Q95QR5:Q95QR5_CAEEL	0.26	0.51	0.25	0.64	0.67	0.74

sm (FBgn0003435) associated phenotypes

feeding behavior defective, long lived, neuroanatomy defective, partially lethal - majority die, short lived, some die during pupal stage

(Information from FlyBase)

Catenin beta-1

Best gene implication in ageing for this target family came from gene P35222 via mapping the annotation from Ensembl ENSG00000168036 via mapping the annotation from EntrezGene 1499 via

mapping the annotation from GenAgeHuman 0223 annotated in GenAge release 17.

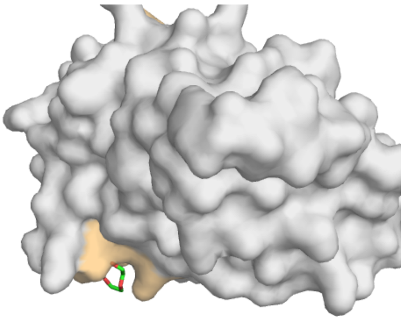

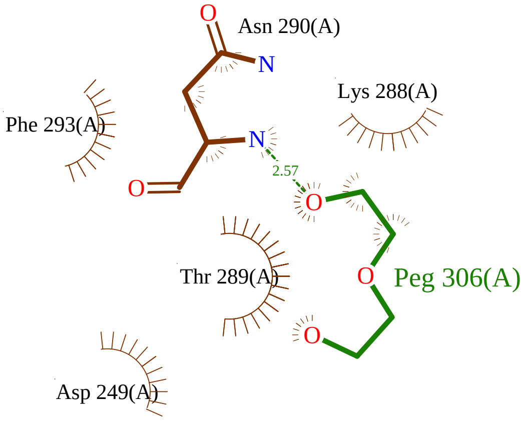

protein	amino acids contacts (binding site)
PDB:3sl9:chainA:P35222	D M K T N F
tr:B4DGU4:B4DGU4_HUMAN	D M K T N F
sp:P35222:CTNB1_HUMAN	D M K T N F
sp:Q9WU82:CTNB1_RAT	D M K T N F
tr:Q80VE7:Q80VE7_MOUSE	D M K T N F
tr:E9Q6A9:E9Q6A9_MOUSE	D M K T N F
sp:Q02248:CTNB1_MOUSE	D M K T N F
sp:P18824:ARM_DROME	E M R N N F

	whole protein		domain-based		contact-based	
protein	ident	simil	ident	simil	ident	simil
PDB:3sl9:chainA:P35222	1.0	1.0	1.0	1.0	1.0	1.0
tr:B4DGU4:B4DGU4_HUMAN	0.99	0.99	1.0	1.0	1.0	1.0
sp:P35222:CTNB1_HUMAN	1.0	1.0	1.0	1.0	1.0	1.0
sp:Q9WU82:CTNB1_RAT	1.0	1.0	1.0	1.0	1.0	1.0
tr:Q80VE7:Q80VE7_MOUSE	0.71	0.71	0.82	0.82	1.0	1.0
tr:E9Q6A9:E9Q6A9_MOUSE	0.74	0.76	0.82	0.83	1.0	1.0
sp:Q02248:CTNB1_MOUSE	1.0	1.0	1.0	1.0	1.0	1.0
sp:P18824:ARM_DROME	0.65	0.83	0.79	0.94	0.5	0.79

arm (FBgn0000117) associated phenotypes

RU486 conditional, cell autonomous, cell migration defective, cell non-autonomous, cell polarity defective, cell shape defective, germline clone, heat sensitive, hyperplasia, increased cell death, lethal - all die before end of pupal stage, memory defective, mitotic cell cycle defective, neuroanatomy defective, partially lethal - majority die, planar polarity defective, segment polarity phenotype, size defective, somatic clone, some die during pupal stage

(Information from FlyBase)

arm (UniProt:P18824) annotation

Function: Isoform neural may associate with CadN and participate in the transmission of developmental information. Can associate with alpha-catenin. Isoform cytoplasmic accumulates through wg signaling; arm function in wg signal transduction is required early in development for determination of neuroblast fate. Arm and Abl proteins function cooperatively at adherens junctions in both the CNS and epidermis. (PubMed:9635189).
Subunit: Interacts with Mer and Moe at the adherens junction. Interacts with Inx2. (PubMed:15047872, PubMed:8666669).
Subcellular location: Cytoplasm ECO:0000269—PubMed:8666669. Cell membrane (PubMed:8666669); Peripheral membrane protein (PubMed:8666669); Cytoplasmic side (PubMed:8666669). Cell junction, adherens junction Note=Inner surface of cell membrane and adherens junction.
Tissue specificity: Isoform cytoplasmic accumulates at low levels in axons, at high levels in specific cells along the CNS midline and in leg and eye imaginal disks. Isoform neural accumu-

lates in the axon tracts of the CNS. Both isoforms accumulate in the peripheral nervous system. (PubMed:9635189).
Developmental stage: Present at all stages, but reaches the highest levels during early to mid-embryogenesis. Isoform cytoplasmic is the predominant one from the cellular blastoderm stage until germ-band retraction. Isoform neural is first seen after germ band retraction.
Ptm: Phosphorylated on Ser, Thr and Tyr residues. Level of phosphorylation varies both during embryonic development and from embryonic tissue to tissue. Sgg is required for phosphorylation and wg signal negatively regulates arm phosphorylation. Hypophosphorylated form of arm increases in steady-state levels. (PubMed:18327897, PubMed:7529201).

(Information from UniProt)

Tyrosine-protein phosphatase non-receptor type 11

Best gene implication in ageing for this target family came from gene G5EC24 via mapping the annotation from WormBase WBGene00004214 annotated in WormBase 2014-03-11. Annotation GO 8340 (determination of adult lifespan) was Inferred from Mutant Phenotype

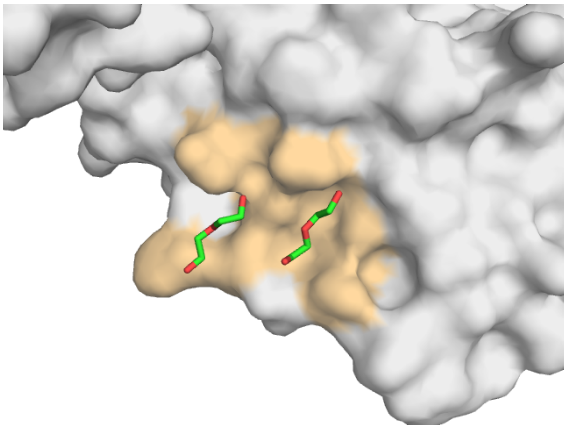

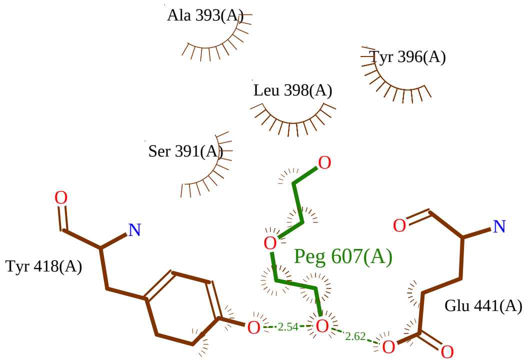

protein	amino acids contacts (binding site)
PDB:4gwf:chainA:Q06124	S A H Y L Y D E
sp:Q06124:PTN11_HUMAN	S A H Y L Y D E
sp:P41499:PTN11_RAT	S A H Y L Y D E
sp:P35235:PTN11_MOUSE	S A H Y L Y D E
sp:P29349:CSW_DROME	N T S Y L Y N D
tr:G5EC24:G5EC24_CAEEL	R V Q Y L L N D

	whole protein		domain-based		contact-based	
protein	ident	simil	ident	simil	ident	simil
PDB:4gwf:chainA:Q06124	1.0	1.0	1.0	1.0	1.0	1.0
sp:Q06124:PTN11_HUMAN	1.0	1.0	1.0	1.0	1.0	1.0
sp:P41499:PTN11_RAT	0.99	1.0	0.99	1.0	1.0	1.0
sp:P35235:PTN11_MOUSE	0.99	1.0	1.0	1.0	1.0	1.0
sp:P29349:CSW_DROME	0.41	0.57	0.39	0.57	0.38	0.72
tr:G5EC24:G5EC24_CAEEL	0.32	0.64	0.37	0.66	0.25	0.7

csw (FBgn0000382) associated phenotypes

dominant, germline clone, heat sensitive, increased cell death, lethal - all die before end of pupal stage, maternal effect, rescuable maternal effect, somatic clone, some die during pupal stage

(Information from FlyBase)

csw (UniProt:P29349) annotation

Function: Required in all receptor tyrosine kinase signaling pathways. Functions downstream of the receptor tyrosine kinase torso, acting in concert with D-Raf via tailless. Also functions down-

stream of Egfr (epidermal growth factor receptor) and btl (fibroblast growth factor receptor). The SH2 domain suggests that csw effects its role by mediating heteromeric protein interactions. Maternally required for normal determination of cell fates at the termini of the embryo. Required for cell fate specification of the ventral ectoderm, in the developing embryonic CNS and for embryonic tracheal cell migration. Functions during imaginal development for proper formation of adult structures such as eyes, aristae, L5 wing vein and the tarsal claw. (PubMed:1638629, PubMed:8948575).
Subcellular location: Cytoplasm.
Tissue specificity: Expressed uniformly throughout all tissues during embryogenesis. (PubMed:1638629).
Developmental stage: Expressed throughout development. (PubMed:1638629).
Miscellaneous: The PTPase domain is interrupted by a PTPase insert which shares no homologies with other PTPase proteins. This PTPase insert is reminiscent of the kinase insert within the kinase catalytic domains of several receptor tyrosine kinases.

(Information from UniProt)

ptp-2 (UniProt:G5EC24) annotation
Subcellular location: Cytoplasm ECO:0000256—PIRNR:PIRNR000929.

(Information from UniProt)

Histone acetyltransferase p300

Best gene implication in ageing for this target family came from gene P34545 via mapping the annotation from WormBase WBGene00000366 annotated in WormBase 2014-03-11. Annotation GO 8340 (determination of adult lifespan) was Inferred from Mutant Phenotype

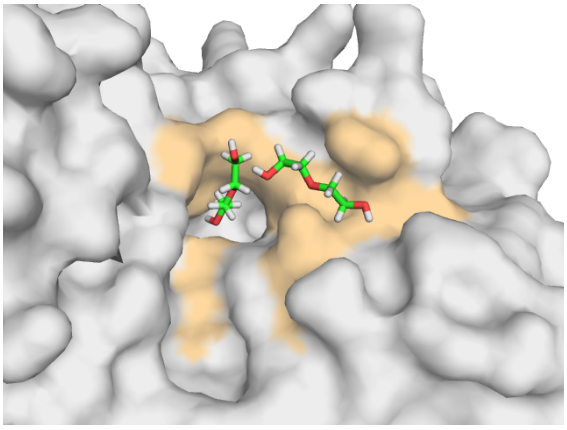

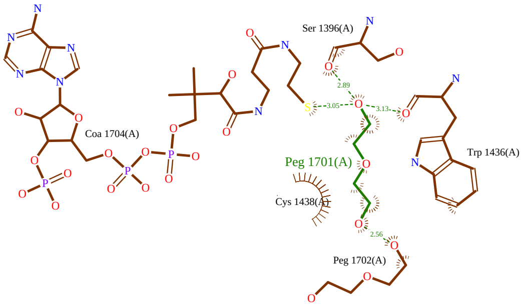

protein	amino acids contacts (binding site)
PDB:3i3j:chainA:Q09472	V W F - - - - - - -
PDB:4pzr:chainA:Q09472	- - - S W C D D Y R
sp:Q09472:EP300_HUMAN	V W F S W C D D Y R
sp:Q92793:CBP_HUMAN	V W F S W C D D Y R
tr:Q91XT0:Q91XT0_RAT	- - - - - - - - - -
sp:B2RWS6:EP300_MOUSE	I W F S W C D D Y R
tr:O01368:O01368_DROME	V W F A W C D D Y R
tr:Q9W321:Q9W321_DROME	V W F A W C D D Y R
sp:P34545:CBP1_CAEEL	C W F A W C D D Y R
sp:P35817:BDF1_YEAST	E R F I D T D A S -

protein	whole protein ident	simil	domain-based ident	simil	contact-based ident	simil
PDB:3i3j:chainA:Q09472	1.0	1.0	1.0	1.0	0.3	0.0
PDB:4pzr:chainA:Q09472	1.0	1.0	1.0	1.0	0.7	0.4
sp:Q09472:EP300_HUMAN	1.0	1.0	1.0	1.0	1.0	1.0
sp:Q92793:CBP_HUMAN	0.56	0.81	0.88	0.97	1.0	1.0
tr:Q91XT0:Q91XT0_RAT	0.14	0.14	0.0	0.0	0.0	0.0
sp:B2RWS6:EP300_MOUSE	0.94	0.98	0.99	1.0	0.9	0.97
tr:O01368:O01368_DROME	0.26	0.54	0.61	0.8	0.9	0.91
tr:Q9W321:Q9W321_DROME	0.26	0.56	0.61	0.8	0.9	0.91
sp:P34545:CBP1_CAEEL	0.27	0.56	0.54	0.84	0.8	0.73
sp:P35817:BDF1_YEAST	0.03	0.15	0.09	0.35	0.2	0.23

nej (FBgn0261617) associated phenotypes

cell migration defective, chemical sensitive, developmental rate defective, dominant, gravitaxis defective, heat sensitive, hypoactive, lethal - all die before end of P-stage, lethal - all die before end of prepupal stage, lethal - all die before end of pupal stage, mitotic cell cycle defective, neuroanatomy defective, neurophysiology defective, partially lethal - majority die, partially lethal - majority live, somatic clone, some die during P-stage, some die during pharate adult stage, some die during prepupal stage, some die during pupal stage

(Information from FlyBase)

cbp-1 (WBGene00000366) associated phenotypes

bag of worms, embryonic lethal, larval lethal, protruding vulva, sick, slow growth, sterile, vulvaless

(Information from WormBase)

cbp-1 (UniProt:P34545) annotation

Function: Acetyltransferase enzyme. Acetylates histones, giving a specific tag for transcriptional activation (By similarity). May prevent DNA damage-induced apoptosis by inhibiting cep-1-dependent transcription activation of the programmed cell death activator egl-1 (PubMed:19521535). (UniProtKB:Q92793, PubMed:19521535).
Subunit: Interacts (via N-terminus domain and HAT domain) with prmt-5; the interaction results in methylation of cbp-1 (PubMed:19521535). Interacts (via HAT domain) with cep-1; cep-1 transcriptional activity may be inhibited by interaction with methylated cbp-1 (PubMed:19521535). Component of a complex that contains prmt-5 and cbp-1 (PubMed:19521535). (PubMed:19521535, PubMed:19521535).
Subcellular location: Nucleus (UniProtKB:Q92793).
Ptm: Methylation by prmt-5 may repress the capacity of cbp-1 to enhance cep-1-dependent transcription of egl-1. (PubMed:19521535).

(Information from UniProt)

Mitogen-activated protein kinase 14

Best gene implication in ageing for this target family came from gene O62618 annotated in UniProt release 2014_02. Annotation GO 8340 (determination of adult lifespan) was Inferred from Mutant Phenotype

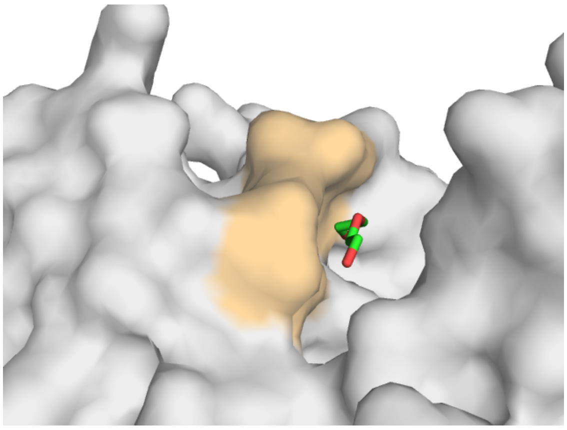

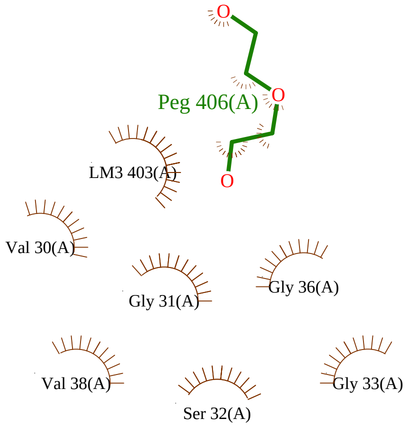

protein	amino acids contacts (binding site)											
PDB:4ewq:chainA:Q16539	V	G	S	G	G	V	Q	N	L	A	Y	A
PDB:4f9y:chainA:Q16539	V	G	S	G	G	V	Q	N	L	A	Y	A
tr:L7RSM2:L7RSM2_HUMAN	V	G	S	G	G	V	Q	N	L	A	Y	A
sp:Q16539:MK14_HUMAN	V	G	S	G	G	V	Q	N	L	A	Y	A
tr:G3V617:G3V617_RAT	V	G	S	G	G	V	Q	N	L	A	Y	A
tr:Q56A33:Q56A33_RAT	V	G	S	G	G	V	Q	N	L	A	Y	A
sp:P47811:MK14_MOUSE	V	G	S	G	G	V	Q	N	L	A	Y	A
tr:Q5U421:Q5U421_MOUSE	V	G	S	G	G	V	Q	N	L	A	Y	A
sp:O62618:MK14A_DROME	V	G	S	G	G	V	Q	N	L	P	Y	E
tr:E1JIV6:E1JIV6_DROME	V	G	S	G	G	V	Q	N	L	P	Y	E
sp:Q17446:PMK1_CAEEL	I	G	T	G	G	V	Q	T	Q	E	Y	A
sp:P32485:HOG1_YEAST	V	G	M	G	G	V	Q	E	D	P	Y	A

	whole protein		domain-based		contact-based	
protein	ident	simil	ident	simil	ident	simil
PDB:4ewq:chainA:Q16539	1.0	1.0	1.0	1.0	1.0	1.0
PDB:4f9y:chainA:Q16539	1.0	1.0	1.0	1.0	1.0	1.0
tr:L7RSM2:L7RSM2_HUMAN	0.96	0.99	0.95	0.99	1.0	1.0
sp:Q16539:MK14_HUMAN	1.0	1.0	1.0	1.0	1.0	1.0
tr:G3V617:G3V617_RAT	0.99	1.0	0.99	1.0	1.0	1.0
tr:Q56A33:Q56A33_RAT	0.95	0.99	0.95	0.99	1.0	1.0
sp:P47811:MK14_MOUSE	0.99	1.0	0.99	1.0	1.0	1.0
tr:Q5U421:Q5U421_MOUSE	0.96	0.99	0.95	0.99	1.0	1.0
sp:O62618:MK14A_DROME	0.67	0.88	0.74	0.91	0.83	0.9
tr:E1JIV6:E1JIV6_DROME	0.67	0.88	0.74	0.91	0.83	0.9
sp:Q17446:PMK1_CAEEL	0.61	0.84	0.71	0.92	0.58	0.71
sp:P32485:HOG1_YEAST	0.41	0.68	0.54	0.85	0.67	0.71

Mpk2 (FBgn0015765) associated phenotypes

heat stress response defective, immune response defective, non-enhancer of variegation, non-suppressor of variegation, nutrition conditional, osmotic stress response defective, oxidative stress response defective, partially lethal - majority die, reduced, short lived, starvation stress response defective, stress response defective

(Information from FlyBase)

Mpk2 (UniProt:O62618) annotation

Function: Kinase involved in a signal transduction pathway. May down-regulate insect immunity gene expression after prolonged infection. (PubMed:9417090, PubMed:9584193).
Cofactor:Mg(2+)
Enzyme regulation: Activated by threonine and tyrosine phosphorylation by Mkk3 in response to environmental stress. (PubMed:9417090, PubMed:9584193).
Subcellular location: Nucleus (PubMed:9417090).
Developmental stage: Expressed both maternally and zygotically. Levels are highest at the pre-

blastoderm stage but low levels are present throughout development. (PubMed:9584193).
Domain: The TXY motif contains the threonine and tyrosine residues whose phosphorylation activates the MAP kinases.
Ptm: Dually phosphorylated on Thr-184 and Tyr-186, which activates the enzyme. (PubMed:9417090).

(Information from UniProt)

pmk-1 (WBGene00004055) associated phenotypes

cell stress response variant, drug induced gene expression variant, gene expression level reduced, lethal, life span variant, organism oxidative stress response hypersensitive, pathogen induced gene expression variant, pathogen susceptibility increased, sterile

(Information from WormBase)

pmk-1 (UniProt:Q17446) annotation

Function: Responds to activation by environmental stress and pro- inflammatory cytokines by phosphorylating downstream targets. Functions downstream of the MAPKK sek-1 and the MAPKKK nsy-1 as the MAP kinase required for pathogen resistance. (PubMed:11703092, PubMed:12142542).
Cofactor: Mg(2+) Evidence=(PubMed:11703092);
Enzyme regulation: Activated by phosphorylation on threonine and tyrosine. Inhibited by pyridinyl-imidazole related compounds. (PubMed:11703092).
Domain: The TXY motif contains the threonine and tyrosine residues whose phosphorylation activates the MAP kinases.
Ptm: Dually phosphorylated on Thr-191 and Tyr-193, which activates the enzyme.

(Information from UniProt)

Peptidoglycan-recognition protein LF

Best gene implication in ageing for this target family came from gene Q8SXQ7 annotated in UniProt release 2014_02. Annotation GO 8340 (determination of adult lifespan) was Inferred from Mutant Phenotype

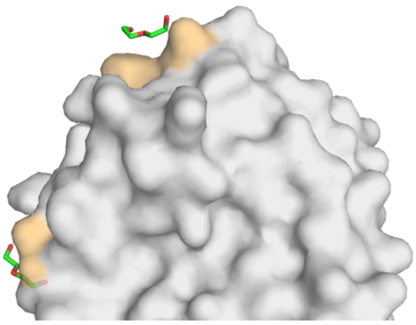

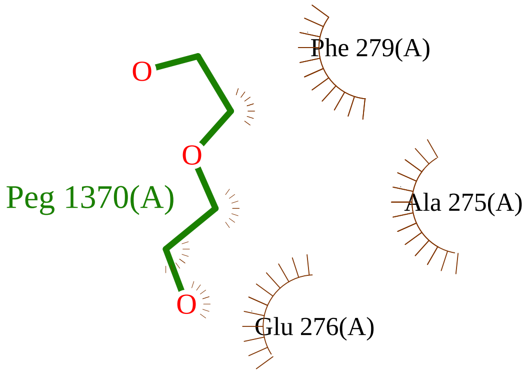

protein	amino acids contacts (binding site)			
PDB:2xz8:chainA:Q8SXQ7	I	A	E	F
sp:Q96PD5:PGRP2_HUMAN	D	-	-	-
tr:D3ZPS3:D3ZPS3_RAT	-	-	-	-
tr:D4AB41:D4AB41_RAT	-	-	-	-
tr:M0R485:M0R485_RAT	-	-	-	-
tr:Q54A77:Q54A77_MOUSE	-	-	-	-
sp:Q8VCS0:PGRP2_MOUSE	-	-	-	-
sp:Q8SXQ7:PGPLF_DROME	I	A	E	F

protein	whole protein ident	simil	domain-based ident	simil	contact-based ident	simil
PDB:2xz8:chainA:Q8SXQ7	1.0	1.0	1.0	1.0	1.0	1.0
sp:Q96PD5:PGRP2_HUMAN	0.09	0.22	0.01	0.12	0.0	0.0
tr:D3ZPS3:D3ZPS3_RAT	0.02	0.04	0.0	0.0	0.0	0.0
tr:D4AB41:D4AB41_RAT	0.01	0.06	0.0	0.0	0.0	0.0
tr:M0R485:M0R485_RAT	0.04	0.11	0.0	0.0	0.0	0.0
tr:Q54A77:Q54A77_MOUSE	0.09	0.21	0.0	0.0	0.0	0.0
sp:Q8VCS0:PGRP2_MOUSE	0.09	0.21	0.0	0.0	0.0	0.0
sp:Q8SXQ7:PGPLF_DROME	1.0	1.0	1.0	1.0	1.0	1.0

PGRP-LF (FBgn0035977) associated phenotypes

immune response defective, long lived, partially lethal - majority die

(Information from FlyBase)

PGRP-LF (UniProt:Q8SXQ7) annotation

Function: Peptidoglycan-recognition protein probably involved in innate immnunity by binding to peptidoglycans (PGN) of bacteria and activating the immune response.
Subcellular location: Membrane ; Multi-pass membrane protein
Induction: Regulated by the imd/Relish pathway. (PubMed:12032070).

(Information from UniProt)

CREB-binding protein

Best gene implication in ageing for this target family came from gene Q92793 via mapping the annotation from Ensembl ENSG00000005339 via mapping the annotation from EntrezGene 1387 via mapping the annotation from GenAgeHuman 0064 annotated in GenAge release 17.

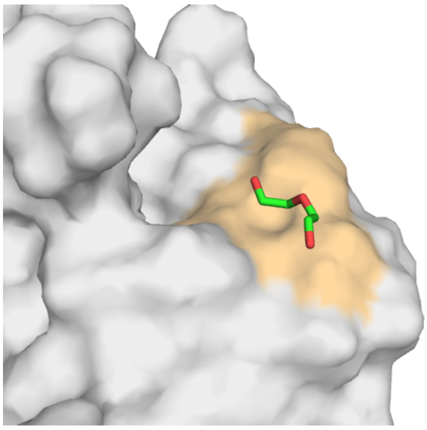

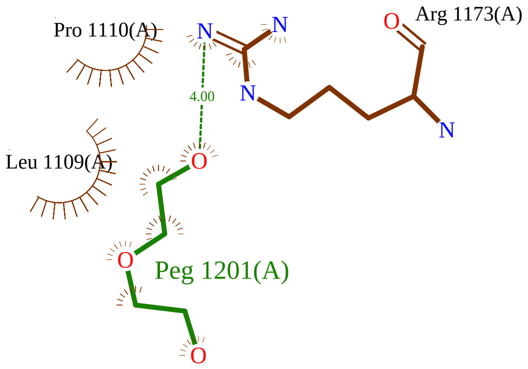

protein	amino acids contacts (binding site)		
PDB:4ouf:chainA:Q92793	L	P	R
sp:Q09472:EP300_HUMAN	L	P	R
sp:Q92793:CBP_HUMAN	L	P	R
tr:F1M9G7:F1M9G7_RAT	L	P	R
tr:Q8QZV8:Q8QZV8_MOUSE	L	P	R
tr:F8VPR5:F8VPR5_MOUSE	L	P	R
tr:Q8T3Z6:Q8T3Z6_DROME	Y	H	P
tr:Q9W1S2:Q9W1S2_DROME	W	V	L
tr:Q4QQ92:Q4QQ92_DROME	W	V	L
tr:Q058W2:Q058W2_DROME	Y	H	P
tr:Q9VCG6:Q9VCG6_DROME	Y	H	P
tr:Q8T3Z8:Q8T3Z8_DROME	L	D	V
tr:Q4V5A7:Q4V5A7_DROME	L	D	V
tr:O17681:O17681_CAEEL	-	-	-
tr:Q9TZB8:Q9TZB8_CAEEL	-	-	-
sp:Q07442:BDF2_YEAST	F	P	E
sp:P35817:BDF1_YEAST	Y	P	I

	whole protein		domain-based		contact-based	
protein	ident	simil	ident	simil	ident	simil
PDB:4ouf:chainA:Q92793	0.97	0.99	1.0	1.0	1.0	1.0
sp:Q09472:EP300_HUMAN	0.59	0.81	0.86	0.96	1.0	1.0
sp:Q92793:CBP_HUMAN	1.0	1.0	1.0	1.0	1.0	1.0
tr:F1M9G7:F1M9G7_RAT	0.96	0.99	0.98	0.99	1.0	1.0
tr:Q8QZV8:Q8QZV8_MOUSE	0.95	0.98	0.98	1.0	1.0	1.0
tr:F8VPR5:F8VPR5_MOUSE	0.96	0.99	0.98	1.0	1.0	1.0
tr:Q8T3Z6:Q8T3Z6_DROME	0.02	0.06	0.29	0.64	0.0	0.52
tr:Q9W1S2:Q9W1S2_DROME	0.02	0.06	0.26	0.57	0.0	0.53
tr:Q4QQ92:Q4QQ92_DROME	0.02	0.07	0.26	0.57	0.0	0.53
tr:Q058W2:Q058W2_DROME	0.02	0.09	0.28	0.63	0.0	0.52
tr:Q9VCG6:Q9VCG6_DROME	0.03	0.12	0.29	0.64	0.0	0.52
tr:Q8T3Z8:Q8T3Z8_DROME	0.02	0.14	0.19	0.7	0.33	0.5
tr:Q4V5A7:Q4V5A7_DROME	0.02	0.13	0.19	0.7	0.33	0.5
tr:O17681:O17681_CAEEL	0.01	0.04	0.0	0.0	0.0	0.0
tr:Q9TZB8:Q9TZB8_CAEEL	0.03	0.07	0.0	0.0	0.0	0.0
sp:Q07442:BDF2_YEAST	0.03	0.15	0.29	0.7	0.33	0.75
sp:P35817:BDF1_YEAST	0.03	0.16	0.29	0.71	0.33	0.55

tbrd-1 (FBgn0039124) associated phenotypes

male fertile

(Information from FlyBase)

NAD-dependent protein deacetylase sirtuin-3, mitochondrial

Best gene implication in ageing for this target family came from gene Q9I7I7 annotated in UniProt release 2014_02. Annotation GO 8340 (determination of adult lifespan) was Inferred from Mutant Phenotype

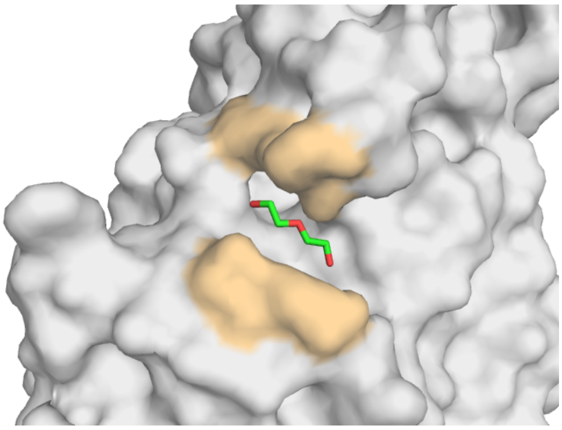

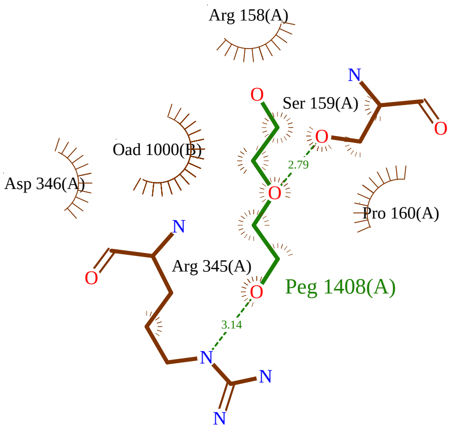

protein	amino acids contacts (binding site)
PDB:4bve:chainA:Q9NTG7	R S P R D G D H
PDB:4bvg:chainA:Q9NTG7	R S P R D G D H
tr:E9PK80:E9PK80_HUMAN	R S P R D G D H
tr:E9PM75:E9PM75_HUMAN	R S P R D G D H
tr:E9PN58:E9PN58_HUMAN	R S P R D G D H
sp:Q9NTG7:SIR3_HUMAN	R S P R D G D H
tr:B2RZ31:B2RZ31_RAT	R S P R D G D Q
tr:C6ZII9:C6ZII9_RAT	R S P R D G D Q
tr:D3YVQ5:D3YVQ5_MOUSE	R S P R D G D H
tr:Q4FJK3:Q4FJK3_MOUSE	R S P R D G D H
sp:Q8R104:SIR3_MOUSE	R S P R D G D H
sp:Q9I7I7:SIRT2_DROME	R S P R D G D A
sp:P53686:HST2_YEAST	R S P L E Q Y E

	whole protein		domain-based		contact-based	
protein	ident	simil	ident	simil	ident	simil
PDB:4bve:chainA:Q9NTG7	1.0	1.0	1.0	1.0	1.0	1.0
PDB:4bvg:chainA:Q9NTG7	1.0	1.0	1.0	1.0	1.0	1.0
tr:E9PK80:E9PK80_HUMAN	0.8	0.8	0.96	0.96	1.0	1.0
tr:E9PM75:E9PM75_HUMAN	0.86	0.86	0.74	0.74	1.0	1.0
tr:E9PN58:E9PN58_HUMAN	0.78	0.79	0.69	0.7	1.0	1.0
sp:Q9NTG7:SIR3_HUMAN	1.0	1.0	1.0	1.0	1.0	1.0
tr:B2RZ31:B2RZ31_RAT	0.55	0.62	0.71	0.82	0.88	0.97
tr:C6ZII9:C6ZII9_RAT	0.63	0.74	0.82	0.95	0.88	0.97
tr:D3YVQ5:D3YVQ5_MOUSE	0.4	0.44	0.41	0.47	1.0	1.0
tr:Q4FJK3:Q4FJK3_MOUSE	0.55	0.62	0.71	0.82	1.0	1.0
sp:Q8R104:SIR3_MOUSE	0.62	0.72	0.82	0.95	1.0	1.0
sp:Q9I7I7:SIRT2_DROME	0.34	0.55	0.41	0.61	0.88	0.9
sp:P53686:HST2_YEAST	0.26	0.49	0.29	0.55	0.38	0.54

Sirt2 (FBgn0038788) associated phenotypes

heat sensitive, lethal - all die before end of pupal stage, some die during pupal stage

(Information from FlyBase)

Sirt2 (UniProt:Q9I7I7) annotation

Function: NAD-dependent protein deacetylase (By similarity). May be involved in the regulation of life span. (, PubMed:17159295).

Cofactor: Zn(2+)Note=Binds 1 zinc ion per subunit. ;

Disruption phenotype: Causes lethality during development. Induced silencing shortens life span. (PubMed:17159295).

(Information from UniProt)
